# Much Higher Case-fatality Rates of Index Cases. Commentary: Differences in the Epidemiology of Human Cases of Avian Influenza A(H7N9) and A(H5N1) Viruses Infection

**DOI:** 10.3389/fpubh.2016.00116

**Published:** 2016-06-20

**Authors:** Xiaodong Zhang

**Affiliations:** ^1^Key Laboratory of Zoonosis Research, Ministry of Education, Institute of Zoonosis, College of Veterinary Medicine, Jilin University, Changchun, China; ^2^National Key Laboratory of Veterinary Biotechnology, Harbin Veterinary Research Institute, Chinese Academy of Agricultural Sciences, Harbin, China

**Keywords:** avian influenza viruses, cluster index cases, sporadic human cases, case fatality rate, adaptation

To date, avian influenza A(H5N1) and A(H7N9) viruses have caused a large number of human infections with high case-fatality rates, and fighting avian influenza viruses (AIVs) seems a protracted war (1). A great health concern has been whether the “lingering” avian influenza A(H5N1) and A(H7N9) viruses would acquire the ability to become easily human transmissible, possibly leading to the emergence of a new influenza pandemic. Clusters of human H5N1 and H7N9 viruses infection have become a serious public health concern as limited human-to-human transmission may occur through close contact in some clusters (2, 3).

I read with great interest the article by Qin et al. for their systematic study on the comparison of characteristics (e.g., median age and case fatality risk) of sporadic and cluster cases of human H5N1 and H7N9 viruses infection (4). In their study, in order to examine whether the individual characteristics of cluster cases are systematically different from other cases, they actually compared cluster secondary cases with the combined group of sporadic cases and the index case of each cluster (sporadic/index cases), as the authors thought that index case in each cluster was essentially detected in the same way as sporadic human cases. Index case in each cluster emerges spatially and temporally not fundamentally different from sporadic cases, so often cluster index cases and sporadic cases are treated identically. However, these do not necessarily mean that index cases possess the same epidemiological characteristics as sporadic cases do. Cluster index case is the initial patient in each cluster after all and ought to be differentiated from sporadic cases when examining the possible differences in the characteristics between sporadic cases and cluster cases, in the event that index cases possess distinctive features. In this commentary, I would like to add complementary data analysis on cluster index cases of human H5N1 and H7N9 viruses infection and make comparisons of epidemiological characteristics between index cases and other cases.

As shown in Table 1, the median age of H7N9 cluster index cases (46 years) was obviously higher than H7N9 cluster secondary cases (31 years) and, meanwhile, was obviously lower than H7N9 sporadic/index cases (59 years). Furthermore, H5N1 infections for cluster index cases were markedly higher among females (64%) than males (36%), which was obviously different from H5N1 cluster secondary cases (females, 48% and males, 52%) and H5N1 sporadic/index cases (females, 54% and males, 46%). Additionally, it needs to be pointed out that the difference between index cases and sporadic cases would be larger than the difference between index cases and sporadic/index cases (see detail explanations in Author Notes), but not much larger because index cases occupy only a small proportion in the combined group – sporadic/index cases (see sample numbers in Table 1).

**Table 1:** **Demographic characteristics and outcomes of cluster index, cluster secondary, and sporadic cases of human H5N1 and H7N9 viruses infection**.^a^

Characteristics	H5N1 cases				H7N9 cases			
	Total (*n* = 720)	Cluster index cases (*n* = 55)	Cluster secondary cases (*n* = 89)	Sporadic cases/cluster index cases (*n* = 631)	Total (*n* = 460)	Cluster index cases (*n* = 16)	Cluster secondary cases (*n* = 20)	Sporadic cases/cluster index cases (*n* = 440)
**Age**
Median (range)	18 (0.3, 86)	15 (0.3, 52)	16 (1, 51)	18 (0.7, 86)	58 (0.4, 91)	45.5 (2, 61)	31 (3, 87)	59 (0.4, 91)
**Age group**
0–9	232 (33.4%)	14 (25.5%)	30 (34.1%)	201 (33.1%)	25 (5.4%)	2 (12.5%)	7 (35.0%)	18 (4.1%)
10–19	138 (19.9%)	21 (38.2%)	21 (23.9%)	118 (19.4%)	4 (0.9%)	0 (0.0%)	0 (0.0%)	4 (0.9%)
20–29	150 (21.6%)	9 (16.4%)	23 (26.1%)	127 (20.9%)	18 (3.9%)	1 (6.3%)	2 (10.0%)	16 (3.6%)
30–39	110 (15.8%)	8 (14.5%)	12 (13.6%)	98 (16.1%)	61 (13.3%)	4 (25.0%)	3 (15.0%)	58 (13.2%)
40–49	34 (4.9%)	2 (3.6%)	1 (1.1%)	33 (5.4%)	44 (9.6%)	1 (6.3%)	1 (5.0%)	43 (9.8%)
50–59	20 (2.9%)	1 (1.8%)	1 (1.1%)	19 (3.1%)	91 (19.8%)	6 (37.5%)	4 (20.0%)	87 (19.8%)
≥60	11 (1.6%)	0 (0.0%)	0 (0.0%)	11 (1.8%)	217 (47.2%)	2 (12.5%)	3 (15.0%)	214 (48.6%)
Unknown	25	0	1	24	0	0	0	0
**Gender**
Female	370 (53.1%)	35 (63.6%)	43 (48.3%)	328 (53.9%)	145 (31.5%)	5 (31.25%)	9 (45.0%)	136 (30.9%)
Male	327 (46.9%)	20 (36.4%)	46 (51.7%)	280 (46.1%)	315 (68.5%)	11 (68.75%)	11 (55.0%)	304 (69.1%)
Unknown	23	0	0	23	0	0	0	0
**Outcome**
Death	426 (59.8%)	44 (83.0%)	45 (54.9%)	381 (61.6%)	180 (40.6%)	9 (64.3%)	4 (26.7%)	175 (41.2%)
Survival	286 (40.2%)	9 (17.0%)	37 (45.1%)	248 (39.4%)	263 (59.4%)	5 (35.7%)	11 (73.3%)	250 (58.8%)
Unknown	8	2	7	2	17	2	5	15

*^a^Original information of “Cluster Index Cases” and “Cluster Secondary Cases” of human H5N1 and H7N9 viruses infection were extracted from the supplementary materials of Qin et al.’s article (4) and then processed. However, the information on each sporadic case was not published in the supplementary materials, so statistical analysis between sporadic cases and cluster index cases could not be performed, the data of “Total” in Qin et al.’s article had to be reused in this Table 1, and also the data of “Sporadic/Cluster Index Cases” in Qin et al.’s article had to be reused in this Table 1 as “spare tire” for “Sporadic Cases.” Despite the impossibility of performing statistical analysis, both 23% higher CFRs of cluster index cases of H5N1 and H7N9 viruses infection (as compared with the respective CFRs of all symptomatic H5N1 and H7N9 cases), actually surpassing the difference in the CFRs of all symptomatic H5N1 and H7N9 cases, do matter*.

Very surprisingly, an important characteristic commonly shared by cluster index cases of H5N1 and H7N9 viruses infection was discovered. The case fatality rates (CFRs) of cluster index cases of H5N1 and H7N9 were unexpectedly high (83% and 64%, respectively), over 20% higher than the respective CFRs of sporadic/index cases of H5N1 and H7N9 (62% and 41%) and naturally much higher than the respective CFRs of sporadic cases of H5N1 and H7N9 (see detail explanations in Author Notes), thus suggesting higher virulence for the causative agents of clusters than the agents of sporadic cases overall. Moreover, the CFRs of cluster index cases of H5N1 and H7N9 (83% and 64%) were also 23% higher than the respective CFRs of all symptomatic H5N1 and H7N9 cases (60% and 41%). In fact, the difference in the CFRs between index cases and respective sporadic cases of human H5N1 or H7N9 infection is larger than the difference in the CFRs between all symptomatic H5N1 and H7N9 cases which is close to 20% (the CFR of all symptomatic cases for a disease that causes mortality is an important epidemic parameter, representing a measure of the severity of the disease), thus deserving critical attention.

What is the significance of much higher CFRs of cluster index cases than respective sporadic cases of H5N1 and H7N9 viruses infection? It reminds me of a traditional method to produce live-attenuated vaccines, such as attenuated lapinized Chinese strain of classical swine fever virus (5); during serial passage in non-susceptible hosts, the pathogen would gradually become more adapted to and increase virulence in the new non-susceptible hosts, thus decreasing in virulence with respect to the original host (6). So, viral adaptation to new host may be closely correlated with the enhancement of viral virulence. When AIVs transmission crosses species, much higher CFRs of cluster index cases than respective sporadic cases of H5N1 and H7N9 viruses infection may indicate that overall, the AIVs causing H5N1 and H7N9 clusters exhibit better adapted to human hosts than the AIVs causing sporadic cases, respectively. Finally, data analysis on cluster index cases of H5N1 and H7N9 viruses infection, perhaps more important than on cluster secondary cases, is an unrecognized but invaluable asset to us in the effort to understand clusters of human H5N1 and H7N9 viruses infection.

## Author Notes

The information on each sporadic human case was not published in the supplementary materials of Qin et al.’s article (4), so the data of “Sporadic/Cluster Index Cases” in their paper has to be reused in this Table 1 as “spare tire” for “Sporadic Cases.” Furthermore, it needs to be pointed out that the real differences between cluster index and sporadic cases of human H5N1 and H7N9 viruses infection would not be lessened as compared to the differences between cluster index and sporadic/index cases. An example can be given to explain it: selecting out some students in a class whose average height is higher than the average height of the whole class, will inevitably result in a fact that the average height of the rest (unselected) is lower than the average height of the whole class; then, the difference of average height between those selected students and the rest (unselected) is inevitably larger than the difference of average height between those selected students and the whole class. Alternatively, selecting out some students in a class whose average height is lower than the average height of the whole class will inevitably result in a fact that the average height of the rest (unselected) is higher than the average height of the whole class; then, the difference of average height between those selected students and the rest (unselected) is also inevitably larger than the difference of average height between those selected students and the whole class.

## Author Contributions

The author confirms being the sole contributor of this work and approved it for publication.

## Conflict of Interest Statement

The author declares that the research was conducted in the absence of any commercial or financial relationships that could be construed as a potential conflict of interest.
